# Intra-Articular Injection of Bupivacaine and Adrenaline Reduces Intraoperative and Postoperative Blood Loss in Total Knee Arthroplasty: A Retrospective Case-Control Study

**DOI:** 10.3390/clinpract15050096

**Published:** 2025-05-20

**Authors:** Ahmad Biadsi, Mor Bracha Akselrad, David Segal, Shanny Gur, Michael Markushevich, Yaron Shraga Brin

**Affiliations:** Department of Orthopedic Surgery, Meir Medical Center, Kfar-Saba 4428164, Israel; ahmad.biadsi@clalit.org.il (A.B.); mor.axelrad@clalit.org.il (M.B.A.); david.segal@clalit.org.il (D.S.); shanny.gur@clalit.org.il (S.G.); michael.markushevich@clalit.org.il (M.M.)

**Keywords:** knee arthroplasty, blood loss, intraoperative complications, postoperative complications, intra-articular injections

## Abstract

**Objective**: To evaluate the effect of preoperative intra-articular injection of bupivacaine and adrenaline (BAD) on blood loss and postoperative hemoglobin levels in primary total knee replacement. **Methods**: We retrospectively assessed 38 consecutive patients who underwent primary total knee arthroplasty at our institution between 2018 and 2019, as performed by two chief orthopedic surgeons. The study group included 22 patients who received an intra-articular injection of 40 mL solution of BAD 0.25% preoperatively. The control group included 16 patients who did not receive the BAD injection preoperatively. Both groups received an IV tranexamic acid (TXA) 1 g treatment prior to the first incision. The posterior capsule and soft tissues were infiltrated after femoral chamfer cuts with a 60 mL BAD solution in both groups. Blood loss was evaluated in all patients by measuring the volume collected in the suction container before the first irrigation and prior to cementation. Additional assessments included the volume of blood drained during the first 24 h postoperatively, as well as changes in hemoglobin levels (delta hemoglobin) 24 h after surgery and at hospital discharge. **Results**: The study and the control groups were similar in age, sex, demographics, and comorbidities. The mean patient age was 71.4 ± 6.5 in the injected group and 70.6 ± 7.5 in the control group. The volume of blood suctioned during surgery was significantly lower in the study group compared to the control group (201 ± 84.3 mL vs. 261.25 ± 83.3 mL; *p* = 0.04). Similarly, the amount of blood drained within the first 24 h postoperatively was also reduced in the study group (204.3 ± 91.1 mL vs. 363.44 ± 131.9 mL; *p* = 0.0001). Ultimately, the decrease in hemoglobin levels from baseline to discharge was less pronounced in the study group compared to the control group (1.7 ± 0.9 g/dL vs. 2.44 ± 1.3 g/dL; *p* = 0.038). **Conclusions:** Intra-articular injection of 40 mL bupivacaine and 0.25% adrenaline solution before skin incision may reduce intraoperative and postoperative blood loss among patients undergoing total knee arthroplasty.

## 1. Introduction

Knee osteoarthritis is a common degenerative joint disease characterized by the breakdown of cartilage in the knee, leading to pain, swelling, stiffness, and reduced mobility. It typically develops gradually due to aging, joint wear and tear, or previous injury. Risk factors include obesity, joint overuse, genetics, and previous trauma. As the disease progresses, it can severely impact daily activities and quality of life. Treatment ranges from lifestyle modifications and physical therapy to medications, injections, and ultimately surgical interventions like total knee arthroplasty in advanced cases [1].

Total knee arthroplasty (TKA) is the definitive treatment for end-stage knee osteoarthritis. It is considered a major orthopedic procedure that can result in significant postoperative pain and blood loss. Studies reported surgeons’ visual estimations of blood loss during TKA ranged from 300 to 2200 mL. These values may be inaccurate considering uncounted hidden blood loss [2,3,4,5]. In the past, approximately 10–40% of patients undergoing TKA required blood transfusion because of acute postoperative anemia [5]. Complications of allogenic blood transfusion include the risk of disease transmission, hemolytic reactions, fluid and hemodynamic overload, acute lung injury, coagulopathy, allergic reactions, and febrile non-hemolytic reactions [5,6,7]. Therefore, reducing perioperative blood loss and allogeneic blood requirements should be of the highest clinical importance [8].

Surgeons have responded to these challenges by reassessing the reasons for transfusion and using a three-phase model to minimize blood loss in TKA: the preoperative phase, the intraoperative phase, and the postoperative phase.

In the preoperative phase, focus should be on correcting anemia, considering autologous blood donation, and holding off antiplatelet and anticoagulant medications prior to surgery by the highest medically permissible interval possible. Some surgeons recommend administering iron, while others advocate for the use of erythropoietin prior to surgery to address anemia and reduce blood transfusions [5].

In the intraoperative phase, intraoperative autologous blood transfusion is one of the available tools to reduce allogeneic blood transfusions. Some have shown that using a tourniquet can reduce blood loss, yet this remains a topic of ongoing debate among surgeons. Tranexamic acid plays an important role in blood conservation strategies during TKA and could be administered either intravenously or topically during the operation [5]. Many researchers have confirmed that fibrin sealant is also associated with the reduction of blood loss in TKA when given topically [9]. Another significant and commonly used method to manage blood loss during surgery is hypotensive anesthesia, which lowers blood pressure through various drugs and techniques. This approach typically maintains an average arterial pressure of 55–60 mm Hg throughout the anesthesia period. The goal of this technique is to decrease peripheral blood flow and minimize blood loss during surgery. Additionally, it enhances the clarity of the surgical field while maintaining normal central venous pressure, stroke volume, and cardiac output [10].

Postoperative strategies include cell salvage, reinfusion drain, avoiding intra-articular drainage, and tranexamic acid (TXA) administration perioperatively [4,6,11,12,13,14,15,16,17,18,19,20].

Various hemostatic agents have been used to reduce blood loss intraoperatively, including TXA, TXA plus diluted adrenaline (TXA + DAD), platelet-rich plasma (PRP), and fibrin sealant [13,14,15,16,17]. Several studies have demonstrated the efficiency of TXA and TXA + DAD in decreasing blood loss perioperatively [2,17]. Some reports have shown that the administration of DAD intraoperatively, in various multi-agent model combinations, provided additional benefits. The infiltration method and timing in these reports varied, ranging from preoperative lavage or peri-articular/intra-articular infiltration, including mid-operation infiltration or lavage. Some studies also included postoperative lavage via a drain [5,12,13,14,15,16,17,18,20,21].

This study aimed to evaluate blood loss following TKA with the pre-incision injection of a solution containing bupivacaine and 0.25% adrenaline (BAD). We believe that the administration of BAD offers two primary benefits: it provides an analgesic effect and reduces blood loss. We hypothesized that these effects could enhance the efficacy of TXA alone.

Between 2018 and 2019, the pre-incision injection of the BAD solution was incorporated into our institution’s standard surgical protocol. We hypothesized that combining tranexamic acid (TXA) with the BAD solution administered before the skin incision would result in reduced blood loss.

## 2. Materials and Methods

The study was conducted according to the guidelines of the Declaration of Helsinki and approved by the Institutional Review Board of Meir Medical Center, Kfar-Saba, Israel (approval number MMC-0053-22).

This retrospective case-control study included 38 consecutive patients who underwent primary TKA by the same two chief surgeons in our institution between 2018 and 2019.

### 2.1. Study Group

The study group included 22 patients who received an intra-articular injection of 40 mL BAD solution prior to skin incision.

### 2.2. Control Group

The control group consisted of 16 patients who did not receive the intra-articular BAD solution prior to skin incision.

### 2.3. Inclusion Criteria

Subjects who underwent elective primary TKA for degenerative knee osteoarthritis. All the patients who underwent TKA were assessed preoperatively by laboratory studies, including complete blood count (CBC), biochemistry, and coagulation panel status of INR/PT/PTT. Only patients within the normal range of these studies continued to undergo TKA. Our protocol consists of discontinuing the administration of antiplatelet medications at least 7 days before surgery; direct oral anticoagulants (DOACs) are discontinued according to renal function, usually 48–72 h before surgery; and enoxaparin administration is stopped 24 h prior to surgery. All patients were operated on by the same two chief surgeons (YSB and MM), operating similarly. In all cases, spinal anesthesia was administered using 10–12.5 mg of bupivacaine combined with 20 mcg of fentanyl, along with a propofol infusion at 25–75 mcg/kg/min, adjusted based on the patient’s comorbidities and level of cooperation. The primary solution used during anesthesia was lactated Ringer’s, following a targeted fluid management approach. During TKA, we applied goal-directed fluid therapy with balanced crystalloids.

#### Exclusion Criteria

Patients who were diagnosed with an infection, patients undergoing revision surgery or post-traumatic primary TKA, patients with impaired coagulation diseases, and patients who suffered a previous thromboembolic event.

### 2.4. Pre-Incision Intra-Articular Injection

In 22 patients (the study group), 40 mL of BAD solution was injected intra-articularly prior to skin incision by the same two chief surgeons. In the control group, the 40 mL BAD solution was not administered prior to the skin incision.

The BAD solution was injected intra-articularly in the operating room after prepping. The knee was held in 90 degrees flexion, and an 18-gauge spinal needle was inserted 1 cm below and 1 cm laterally to the inferior pole of the patella at 45 degrees, aiming at the center of the knee. Skin incision was usually performed five minutes following the injection, after draping.

### 2.5. Implants

In all patients, we used the Corin Unity Knee™ Total Knee System, Corin, Cirencester, UK. This system is a fixed-bearing total knee replacement designed to preserve the medial joint line and provide ligament isometry throughout the range of motion.

### 2.6. Surgical Technique

The same two chief surgeons, utilizing similar techniques, led all the cases in a consecutive, unblinded manner. All patients were operated on under spinal anesthesia and treated with IV beta blockers when needed to retain control of permissive hypotension. A prophylactic pre-incision single dose of IV antibiotics and 1 g of IV TXA was given to both groups. No tourniquets were used at any time during the operation in either group. The procedure typically follows these steps:

A standard midline skin incision and a medial parapatellar arthrotomy are used to expose the knee joint.

#### 2.6.1. Bone Resection

Tibia: The proximal tibial cut is made perpendicular to the mechanical axis, using an extramedullary guide. The resection aims to preserve as much bone as possible while ensuring a flat surface for the tibial component.

Femur: A first minimal distal femoral cut is performed, using an intramedullary alignment guide (Corin Unity Knee™ Total Knee System, Corin, Cirencester, UK). Then, a second distal cut is performed using the UNITY-specific tensioners to respect the soft tissues surrounding the knee, which dictate the degree to which the distal cut is performed.

Chamfer cuts: These femoral cuts are made according to the selected rotation, which is determined by the soft tissues using the UNITY-specific tensioners as a guide.

Following chamfer cuts posterior capsule and soft tissues, infiltration with 60 mL of BAD solution in both groups. Local infiltration points include the quadriceps mechanism, the medial gutter, and the pes anserine.

#### 2.6.2. Trialing

Trial femoral, tibial, and polyethylene components are inserted. Knee stability, range of motion, patellar tracking, and overall alignment are evaluated.

#### 2.6.3. Patella Management

UNITY components (Corin Unity Knee™ Total Knee System, Corin, Cirencester, UK) are designed to optimize patellar tracking with or without resurfacing. We chose not to resurface the patella when needed; patellar osteophytes were removed.

#### 2.6.4. Implantation

Final components (Corin Unity Knee™ Total Knee System, Corin, Cirencester, UK) are implanted, typically with cement fixation.

#### 2.6.5. Closure

The wound is closed in layers. The quadriceps tendon and arthrotomy are repaired carefully to allow for early mobilization.

An intra-articular injection of 3 g of TXA was given to both groups after capsule closure. A drain was inserted for all patients prior to skin closure. Low molecular weight heparin (enoxaparin 40 mg) was administered subcutaneously once a day for 14 days postoperatively, starting 12 h after surgery, to all patients.

### 2.7. Perioperative Evaluation

Intraoperative blood loss was estimated by three parameters, all assessed by the two chief surgeons:

1. The Amount of blood collected from the calibrated suction canister before the cementation phase and prior to the joint lavage. It was registered by the rotating nurse in the operating room.
2. The volume of blood drained within 24 h postoperatively was assessed using drains inserted after the surgical procedure, prior to wound closure. Blood loss was collected in calibrated canisters over the 24-h period. Drain removal was performed at 24 h postoperatively by one of two chief surgeons, who also oversaw the evaluation, measurement, and documentation processes.
3. Blood samples were collected by a phlebotomist on the first postoperative day and typically on the third postoperative day, which was the day of discharge. The two lead surgeons evaluated the changes in hemoglobin levels between the preoperative period and 24 h postoperatively, as well as between the preoperative period and discharge.

Patients were encouraged to initiate ambulation as early as possible, ideally on the day of surgery, depending on their recovery from anesthesia. Once movement and sensation in their legs were adequately restored, they began mobilization with the support of a walker and under the direct supervision of a physiotherapist. The physiotherapist provided individualized guidance to ensure safe and effective mobilization, helping patients regain confidence, improve circulation, and reduce the risk of postoperative complications such as deep vein thrombosis and joint stiffness. Early ambulation was considered a critical component of the postoperative rehabilitation protocol aimed at promoting faster recovery and enhancing overall functional outcomes.

### 2.8. Data Analyses

Descriptive statistics were used to present raw data. After confirming normal distribution of the continuous variables with Kolmogorov–Smirnov and Shapiro–Wilk tests, univariate analysis was conducted. A student *t*-test was used to compare continuous variables, and a chi-square test was used to compare categorical variables. Effect size (ES) was calculated by subtracting the mean results in the control group from the mean result in the study group and dividing the result by the standard deviation of the study group measure. ES of 0.2, 0.5, and 0.8 were considered small, medium, and large, respectively. An alpha of 0.05 and a beta of 0.2 were defined for statistical significance. A post-hoc power analysis showed that with the current study patient population, a difference of 1.18 g/dL could be detected with statistical significance, applying an alpha of 0.05 and a beta of 0.02. This difference was slightly lower than one standard deviation of the mean baseline hemoglobin level. If variables have low variance, an even milder difference will be detectable with statistical significance. SPSS version 28.0 software (IBM Corp., Armonk, NY, USA) was used for analysis.

## 3. Results

A total of 38 patients were included in the study. The mean patient age was 71 years in both groups (71.4 ± 6.5 years in the study group and 70.6 ± 7.5 years in the control group). The distribution of sexes was similar between the groups; the study group consisted of 19 females and 3 males, a total of 22 patients. In the control group, there were 12 females and 4 males, a total of 16 patients. The comorbidities between the groups were similar (Table 1).

The amount of blood that was suctioned during surgery and the amount of blood output that was collected by the drain during the first postoperative 24 h were both smaller in the study group compared to the control group (206.1 ± 84.3 vs. 261.3 ± 72.7, ES = 0.65, *p* = 0.042, and 204.3 ± 91.1 vs. 363.4 ± 131.9, ES = 1.74, *p* = 0.0001, respectively). In both groups, no patient required a blood transfusion after the operation. The decrease in hemoglobin levels from baseline to discharge was milder in the study group (−1.7 ± 0.9 g/dL vs. −2.44 ± 1.3 g/dL, ES = 0.82, *p* = 0.038) (Table 2). 

## 4. Discussion

The most significant finding of this study was the confirmation of our hypothesis that intra-articular injection of BAD solution before skin incision in TKA reduces perioperative blood loss.

Over the last decade, many studies have investigated the effect of local and systemic single or multimodal drug combinations on the outcomes of TKA surgeries [3,13,14,17]. Reducing perioperative blood loss is a primary target of interest [21,22].

Studies have shown that the administration of local and systemic TXA before, during, and after TKA surgeries has a positive effect on reducing perioperative blood loss [13,14].

Several studies have investigated the isolated effect of solutions containing bupivacaine and adrenaline on blood loss [12,14,15,16,17,18]. Studies have been conducted to demonstrate the reduction of blood loss with the administration of intra-articular adrenaline. Lombardi et al. found a significantly less pronounced reduction in hemoglobin among patients who received intra-articular soft tissue injection of adrenaline, bupivacaine, and morphine [17]. Gasparine et al. also demonstrated a significant reduction in blood loss after local application of diluted adrenaline and saline solution before tourniquet release [15]. Anderson et al. showed similar results when patients received bupivacaine, adrenaline, and saline solutions divided into pre-skin incision and intraoperative pericapsular infiltrations [12]. Some other studies reported additional benefits of reduced blood loss when utilizing a diluted solution of both adrenaline and TXA compared to TXA alone [14,21,23]. Bhutta et al. demonstrated a significant reduction in blood loss among patients who received local anesthesia infiltration containing adrenaline. They observed a higher likelihood of blood transfusion in those who did not receive this treatment [13]. Nonetheless, some studies have demonstrated no differences in blood loss when using adrenaline-containing solutions during TKA. Malone et al., in a retrospective study of 189 consecutive patients, showed no benefit for diluted adrenaline saline solution lavage following component implantation [18]. Similar results were reported by Mesa-Ramos et al., concluding that local administration of adrenaline into the surgical field did not result in any reduction in blood loss, nor did it modify transfusion requirements [19]. We believe that the discrepancies in findings compared to our study can be attributed to the timing of adrenaline administration, as it was applied at the end of the procedure instead of prior to the skin incision. Our study indicates that preoperative intra-articular injection of the BAD solution provides an additional benefit in reducing blood loss compared to the administration of TXA alone. Blood loss was assessed at three key stages during the procedure, as follows:

1. The volume of blood suctioned from the operative field prior to the first irrigation and cementation. Notably, the difference between the two groups was even more pronounced than reported, as the collected fluids in the injected group included an additional 40 mL from the BAD solution.
2. The study group exhibited a smaller volume of blood drained during the first 24 h postoperatively.
3. Most importantly, the decrease in hemoglobin levels at discharge (delta hemoglobin) was significantly lower in the study group.

No complications, adverse effects, or need for blood products perioperatively were observed in patients from either group of our study.

We encountered no difficulties in incorporating BAD injections into routine practice, given their low cost and the apparent benefits.

There are limitations to this retrospective study. The most obvious limitation is the small number of patients included in our study. On the other hand, achieving statistical differences in a small sample indicates that the results are robust. Another drawback of our study was that it was unblinded and executed in a single center. In our study, we did not collect patients’ data related to postoperative pain control with the intra-articular BAD solution. Several studies suggested improved pain management in the postoperative period with similar methods [17,24]. This can potentially increase the patient’s ability to participate in physical therapy, reduce narcotic need, and possibly shorten the hospitalization period after surgery. We also did not assess variables such as fluid therapy and anesthesia in the postoperative period. Another important drawback is the lack of blinding.

To strengthen and validate our results and theory, more studies should be conducted, preferably multicenter, prospective, double-blind studies, and with a larger study group.

## 5. Conclusions

Our study shows that intra-articular injection of BAD before skin incision in TKA reduces both intraoperative and postoperative blood loss. We recommend the administration of BAD solution in primary TKA prior to the first skin incision. Implementing our conclusions and adopting our approach can help reduce blood loss and enhance the outcomes of total knee arthroplasties.

## Figures and Tables

**Table 1:** Patient demographics and comorbidities.

Variable	Injection *n* = 22	No Injection *n* = 16	*p*-Value
Age, years; mean ± SD	71.4 ± 6.5	70.6 ± 7.5	0.759
Gender, Female/male	19/3	12/4	0.372
Hypertension, *n* (%)	11 (47.8)	9 (56.3)	0.748
COPD, *n* (%)	3 (13)	3 (18.8)	0.674
Diabetes mellitus, *n* (%)	10 (43.5)	6 (37.5)	0.752
Ischemic heart disease, *n* (%)	3 (13)	1 (6.3)	0.631
Atrial fibrillation, *n* (%)	2 (9.5)	0	0.495
Mental disorder, *n* (%)	3 (18)	2 (12.5)	1.00
Osteoporosis, *n* (%)	4 (17.4)	1 (6.3)	0.631
CVA, TIA, *n* (%)	3 (13)	0	0.255
Renal failure, *n* (%)	2 (8.7)	1 (6.3)	1.00
Gastrointestinal disorder, *n* (%)	3 (13)	2 (12.5)	1.00
Thyroid disorder, *n* (%)	0	3 (18.8)	0.061

Legend for Table 1: SD—standard deviation, COPD—Chronic Obstructive Pulmonary Disease, CVA—Cerebrovascular Accident, TIA—Transient Ischemic Attack.

**Table 2:** Results.

Variable *	BAD Injection *n* = 22	No Injection *n* = 16	*p*-Value
Hemoglobin before surgery, g/dL	12.9 ± 1.2	13.74 ± 1.3	0.207
Blood suctioned during surgery, mL	206.1 ± 84.3	261.3 ± 72.7	0.042
Blood drainage output 24 h after surgery, mL	204.3 ± 91.1	363.4 ± 131.9	0.0001
Hemoglobin at discharge, g/dL	11.2 ± 1	11.0 ± 1.3	0.599
Delta hemoglobin at discharge, g/dL	−1.7 ± 0.9	−2.44 ± 1.3	0.038

Legend for Table 2: * All values are mean ± standard deviation Hb—Hemoglobin, g—gram, dL—deciliter, mL—milliliter.

## Data Availability

Data are unavailable due to privacy and ethical restrictions, and is recorded in patients’ files.
